# Analgesic Effects of Intra-Articular Bupivacaine/Intravenous Parecoxib Combination Therapy versus Intravenous Parecoxib Monotherapy in Patients Receiving Total Knee Arthroplasty: A Randomized, Double-Blind Trial

**DOI:** 10.1155/2015/450805

**Published:** 2015-06-11

**Authors:** Shih-Jyun Shen, Pei-Yu Peng, Hsiu-Pin Chen, Jr-Rung Lin, Mel S. Lee, Huang-Ping Yu

**Affiliations:** ^1^Department of Anesthesiology, Chang Gung Memorial Hospital, Taoyuan 333, Taiwan; ^2^College of Medicine, Chang Gung University, Taoyuan 333, Taiwan; ^3^Department of Nursing, Chang Gung Memorial Hospital, Taoyuan 333, Taiwan; ^4^Clinical Informatics and Medical Statistics Research Center, Chang Gung University, Taoyuan 333, Taiwan; ^5^Department of Orthopaedic Surgery, Chang Gung Memorial Hospital, Chiayi 613, Taiwan

## Abstract

*Objectives*. The purpose of this double-blind, randomized study was to investigate whether the addition of intra-articular bupivacaine to intravenous parecoxib could improve pain relief in patients undergoing total knee arthroplasty.* Methods*. A total of 36 patients undergoing total knee arthroplasty were enrolled into our study. These patients were randomly allocated either to a placebo-controlled group or study group. Postoperative pain scores and analgesic consumption were evaluated.* Results*. Numeric rating scale (NRS) data of bupivacaine group in postoperative room were significantly lower than that of control group (control group versus bupivacaine group, 7.9 (6.7–9.1) (mean and 95% confidence interval) versus 4.5 (3.2–5.8) (mean and 95% confidence interval), *p* = 0.001). NRS data of bupivacaine group in ward were also significantly lower than that of control group. A significantly lower dose of meperidine was used in the study group postoperatively during the first 24 hours (control group versus bupivacaine group, 3.08 ± 0.80 mg/Kg versus 2.34 ± 0.42 mg/Kg, *p* = 0.001).* Conclusion*. Intra-articular bupivacaine in combination with intravenous parecoxib may improve pain relief and reduce the demand for rescue analgesics in patients undergoing total knee arthroplasty. The trial is registered with Australian New Zealand Clinical Trials Registry (ACTRN12615000463572).

## 1. Introduction

Knee surgeries usually lead to severe postoperative pain [1], which may result in poor joint motion, leading to extended hospitalization days and delayed rehabilitation [2]. Many methods such as drugs, nerve block, and epidural analgesia [2, 3] have been discussed for pain relief.

Among these, nonsteroidal anti-inflammatory drugs (NSAIDs) are commonly used for pain relief [4, 5]. Parecoxib, a cyclooxygenase-2 selective inhibitor, is widely used for postoperative pain relief [6]. However, previous studies have shown that the analgesic effect of parecoxib alone is not adequate in patients undergoing knee surgery [7].

Intra-articular drug injections may reduce pain following joint operation [8]. Furthermore, intra-articular morphine administered can provide pain relief after anterior cruciate ligament reconstruction [9]. Recent studies have shown that intra-articular bupivacaine injection has an analgesic effect after total hip replacement and a good opioid-sparing effect in the first 12 hours following hip surgery [10]. Local anesthetic is injected into the intra-articular cavity after surgical wound closure, spreading into muscle and soft tissue, which can effectively decrease postoperative pain. In view of this, we hypothesized that intra-articular bupivacaine in combination with intravenous parecoxib may improve postoperative pain relief in patients undergoing total knee arthroplasty.

## 2. Material and Methods

### 2.1. Patient Selection

The study was approved by the Institutional Review Board of Chang Gung Medical Foundation prior to the start of the trial. Written informed consent was obtained from every participant. A total of 40 patients (of both sexes) were prospectively included in the study. All patients were scheduled for total knee arthroplasty under general anesthesia between December 2012 and March 2013. The inclusion criterion was patients with knee osteoarthritis who were recommended for total knee arthroplasty. Preoperative evaluation for general anesthesia was performed. Exclusion criteria included neuropathic pain or sensory disorder in the knee requiring surgery, coagulation abnormalities, severe renal or hepatic impairment, and chronic opioid use. The neuropathic pain was detected according to the modified painDETECT questionnaire [11].

### 2.2. Study Setting

All participants were randomly assigned to either the control group (preoperative intravenous parecoxib) or the bupivacaine group (intra-articular bupivacaine combined with preoperative intravenous parecoxib) on the basis of a concealed allocation approach. A computerized random number table with no restrictions on the randomization was used to determine this allocation. Numbered, opaque, sealed envelopes containing the randomization schedule were kept by an investigator who was not an assessor of the study. The envelopes were opened immediately before the intra-articular injection. All patients were blinded to their allocation. Parecoxib 40 mg was injected intravenously 1 hour before operation. All enrolled patients received general anesthesia by means of orotracheal intubation with propofol (2 mg/Kg), fentanyl (2 mcg/Kg), and cisatracurium (0.2 mg/Kg). Anesthesia was maintained with sevoflurane. In all cases, fentanyl was not given during the last 30 minutes of surgery. Surgery procedures were performed by the same orthopedic technique. After the closure of the surgical wound, an intra-articular injection of 0.5% bupivacaine 60 mL (300 mg) or 0.9% normal saline 60 mL was given into the joint space. The bupivacaine and normal saline were prepared by the pharmacy and were exteriorly indistinguishable. Hence, both the patients receiving the intra-articular injection and the doctor attending these patients did not know which drug was injected.

### 2.3. Postoperative Pain Assessment and Analgesic Protocol

After operation, the numeric rating scale (NRS) scores, wherein score 0 denoted no pain and score 10 denoted the worst pain, were used for the first time for pain assessment. If the NRS score exceeded 4, an intramuscular meperidine 50 mg injection was given for pain relief. The frequency of assessment was every 4 hours and an intramuscular meperidine 50 mg injection was given if needed. Data were collected by nursing staff who were unaware of the study.

### 2.4. Outcome Measurement

The primary outcome was the NRS scores after the operation. The secondary outcome was the amount of meperidine use within the first 24 hours after the operation.

### 2.5. Statistical Analysis

Data were collected and expressed as number, percentage, and mean ± standard deviation. The statistical result of pain scores was expressed as mean and 95% confidence interval. Unpaired Student's *t*-test was used for analysis. A *p* value < 0.05 was considered to be statistically significant. All statistical data were analyzed using the SPSS statistical software.

## 3. Results

### 3.1. Patient Characteristics

**Figure 1:**
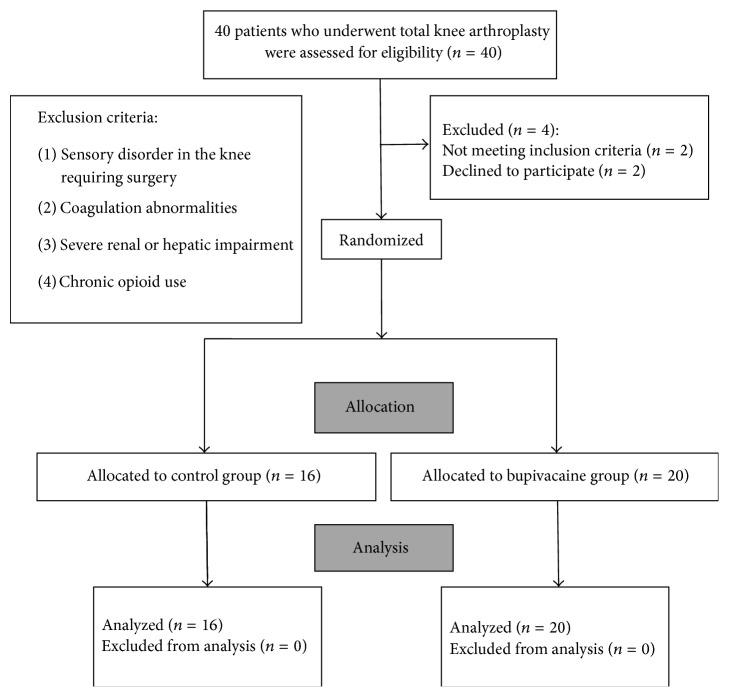
Flow diagram of the study.

Forty patients were enrolled in this study. Four of them were excluded. Two patients declined to participate. One patient had a sensory disorder in the knee and required surgery and one patient had severe renal impairment. The CONSORT flow diagram for the study is shown in Figure 1.

The basic data and preoperative comorbidities of all the patients are shown in Table 1. These patients were similar in age, gender, and American Society of Anesthesiologists (ASA) physical status distribution.

### 3.2. Postoperative Pain Assessment

NRS scores obtained in the postoperative room and in the ward, 24 hours following the operation, are expressed in Tables 2 and 3. NRS data of the bupivacaine group in the postoperative room were significantly lower than those of the control group (control group versus bupivacaine group, 7.9 (6.7, 9.1) versus 4.5 (3.2, 5.8), *p* = 0.001). NRS data of bupivacaine group in the ward were also significantly lower than those of control group (control group versus bupivacaine group, 7.6 (6.4, 8.7) versus 4.5 (3.6, 5.3), *p* = 0.0002).

**Figure 2:**
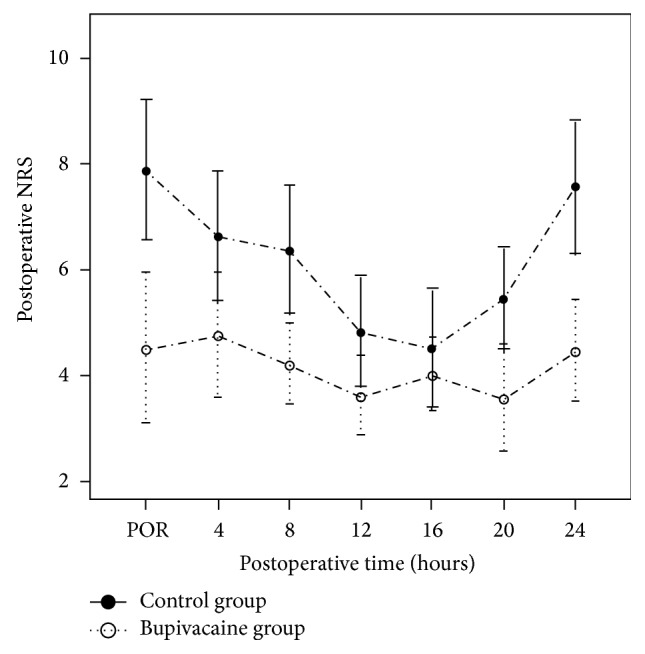
Postoperative numeric rating scale (NRS) scores in the postoperative room (POR) and on the ward. Means and 95% confidence intervals are shown. Scores of the bupivacaine group were lower than those of the control group postoperatively during the first 24 hours. The numbers of patients contributed to each data point were the same as the initial numbers of patients.

Figure 2 shows the postoperative NRS scores. NRS scores of bupivacaine group were significantly lower than those of control group on the first day following the operation.

### 3.3. Postoperative Analgesic Consumption

The total amount of meperidine use is shown in Table 4 (control group versus bupivacaine group, 3.08 ± 0.80 mg/Kg versus 2.34 ± 0.42 mg/Kg, *p* = 0.001), indicating that patients under intra-articular bupivacaine intervention had a lower amount of meperidine use within 24 hours following the operation.

## 4. Discussion

The results of this prospective study suggested that intra-articular bupivacaine (300 mg) in combination with intravenous parecoxib (40 mg) could improve pain relief and reduce meperidine requirements in the management of postoperative pain during the first 24 hours following total knee arthroplasty. Poor control of postoperative pain may lead to a series of adverse effects, including immunosuppression [12, 13]. Multimodal analgesia may achieve optimal analgesia in the management of postoperative pain by reducing opioid consumption and its related adverse events [14, 15]. Recent studies have also shown that multimodal analgesia can improve pain relief following total knee arthroplasty [16, 17]. In addition, our results indicated that patients receiving intra-articular bupivacaine in combination with intravenous parecoxib consumed significantly less meperidine than patients receiving intravenous parecoxib postoperatively in the first 24-hour observation period. The potential advantage of the combination therapy is that patients experience less pain even though they require lower amount of narcotics. Decreased narcotic consumption may decrease the risk of opioid-related adverse events [18–20].

Intra-articular injection of 60 mL of 0.5% bupivacaine was used in the current study. Previous studies have shown that intra-articular infusion of 0.5% bupivacaine at a rate of 2 mL/h for 48 hours produces little or no pain relief [21]. The results suggest that a relatively large bolus injection of bupivacaine may produce better pain relief than a small continuous infusion. The pain relief effect of intra-articular bupivacaine may be associated with the degree of infiltration from the site of injection to the soft tissue around the joint. However, the precise mechanism remains to be determined.

Some limitations of this study should be considered. We did not record mobilization data, and pain scores were determined only at rest. The pain rating at rest alone is not very helpful as it is the functional outcome that is of clinical interest. This includes pain during movement, quadriceps strength, and early ambulation. Evaluation of pain during movement is suggested for further study. Furthermore, a pilot study was not performed to calculate the required sample size. In this study, with such a small sample size, the investigation may not have been sufficiently convincing. In addition, a single-bolus dose of bupivacaine was used in this study. A third group with a higher or lower bupivacaine dose to determine the optimal dose/volume of bupivacaine is suggested for a future study.

## 5. Conclusion

In conclusion, intra-articular bupivacaine in combination with intravenous parecoxib improved pain relief and reduced the demand for rescue analgesics in patients undergoing total knee arthroplasty.

## Figures and Tables

**Table 1:** General characteristics of patients enrolled.

		Control group (*n* = 16)	Bupivacaine group (*n* = 20)	*p*
Characteristic	Age (year)	51.2 ± 13.5	53.1 ± 14.8	0.581
	Male gender	9 (56%)	11 (55%)	0.940

ASA status	II	6 (38%)	8 (40%)	0.878
	III	10 (63%)	12 (60%)	

Preoperative comorbidities	Hypertension	4 (25%)	6 (30%)	0.519
	Diabetes mellitus	4 (25%)	7 (35%)	0.391
	Asthma	1 (6%)	2 (10%)	0.585
	Old cerebral embolism	1 (6%)	1 (5%)	0.698
	Old myocardial infarction	1 (6%)	1 (5%)	0.698

Continuous variables were described as mean ± standard deviation, and the categorical variable was described as number of events (*n*/%); the remaining parameters were compared using an independent *t*-test, and statistical significance was considered when *p* < 0.05. Categorical variables were the number of events (*n*); the Chi-square test was used, and events less than 5 were compared with Fisher's exact test, *p* < 0.05.

**Table 2:** Numeric rating scale (NRS) scores in POR and ward.

	Control group	Bupivacaine group	*p*
NRS in POR	7.9 (6.7, 9.1)	4.5 (3.2, 5.8)	0.001
NRS 24 hours later in ward	7.6 (6.4, 8.7)	4.5 (3.6, 5.3)	0.0002

Continuous variables were described as the mean and 95% confidence interval; an independent *t*-test was used, and statistical significance was considered when *p* < 0.05. POR: postoperative room.

**Table 3:** Postoperative pain score during the first 24 hours.

	NRS score	Time (hours)						
		POR	4	8	12	16	20	24
Control group (*n* = 16)	0–3	2	3	2	5	7	4	0
	4–6	1	1	6	6	6	6	6
	7–10	13	12	8	5	3	6	10

Bupivacaine group (*n* = 20)	0–3	10	5	6	8	8	10	8
	4–6	5	11	12	11	11	7	10
	7–10	5	4	2	1	1	3	2

Numeric rating scale (NRS) scores were divided into 3 groups: mild (NRS score: 0–3), moderate (NRS score: 4–6), and severe (NRS score: 7–10). POR: postoperative room.

**Table 4:** Postoperative meperidine consumption during the first 24 hours.

	Control group	Bupivacaine group	*p*
Postoperative meperidine consumption (mg/Kg)	3.08 ± 0.80	2.34 ± 0.42	0.001

Continuous variables were described as mean ± standard deviation and an independent *t*-test was used; statistical significance was considered when *p* < 0.05.

## References

[1] Vendittoli P.-A., Makinen P., Drolet P. (2006). A multimodal analgesia protocol for total knee arthroplasty: a randomized, controlled study. *The Journal of Bone and Joint Surgery—American Volume*.

[2] Klasen J. A., Opitz S. A., Melzer C., Thiel A., Hempelmann G. (1999). Intraarticular, epidural, and intravenous analgesia after total knee arthroplasty. *Acta Anaesthesiologica Scandinavica*.

[3] Grevstad U., Mathiesen O., Valentiner L. S., Jaeger P., Hilsted K. L., Dahl J. B. (2015). Effect of adductor canal block versus femoral nerve block on quadriceps strength, mobilization, and pain after total knee arthroplasty: a randomized, blinded study. *Regional Anesthesia & Pain Medicine*.

[4] Jirarattanaphochai K., Jung S. (2008). Nonsteroidal antiinflammatory drugs for postoperative pain management after lumbar spine surgery: a meta-analysis of randomized controlled trials. *Journal of Neurosurgery: Spine*.

[5] Abdulla S., Eckhardt R., Netter U., Abdulla W. (2012). Randomized, double-blind, placebo-controlled study to assess the efficacy of nonopioid analgesics on pain following arthroscopic knee surgery. *Pain Research and Treatment*.

[6] Jirarattanaphochai K., Thienthong S., Sriraj W. (2008). Effect of parecoxib on postoperative pain after lumbar spine surgery: a bicenter, randomized, double-blinded, placebo-controlled trial. *Spine*.

[7] Elseify Z. A., El-Khattab S. O., Khattab A. M., Atta E. M., Ajjoub L. F. (2011). Combined parecoxib and I.V. paracetamol provides additional analgesic effect with better postoperative satisfaction in patients undergoing anterior cruciate ligament reconstruction. *Saudi Journal of Anaesthesia*.

[8] Lunn T. H., Husted H., Solgaard S. (2011). Intraoperative local infiltration analgesia for early analgesia after total hip arthroplasty: a randomized, double-blind, placebo-controlled trial. *Regional Anesthesia and Pain Medicine*.

[9] Brandsson S., Karlsson J., Morberg P., Rydgren B., Eriksson B. I., Hedner T. (2000). Intraarticular morphine after arthroscopic ACL reconstruction: a double-blind placebo-controlled study of 40 patients. *Acta Orthopaedica Scandinavica*.

[10] Chen D. W., Hu C.-C., Chang Y.-H., Lee M. S., Chang C.-J., Hsieh P.-H. (2014). Intra-articular bupivacaine reduces postoperative pain and meperidine use after total hip arthroplasty: a randomized, double-blind study. *The Journal of Arthroplasty*.

[11] Hochman J. R., Davis A. M., Elkayam J., Gagliese L., Hawker G. A. (2013). Neuropathic pain symptoms on the modified painDETECT correlate with signs of central sensitization in knee osteoarthritis. *Osteoarthritis and Cartilage*.

[12] Paul J. E., Buckley N., McLean R. F. (2014). Hamilton acute pain service safety study: using root cause analysis to reduce the incidence of adverse events. *Anesthesiology*.

[13] Ahlers O., Nachtigall I., Lenze J. (2008). Intraoperative thoracic epidural anaesthesia attenuates stress-induced immunosuppression in patients undergoing major abdominal surgery. *British Journal of Anaesthesia*.

[14] Nong L., Sun Y., Tian Y., Li H. (2013). Effects of parecoxib on morphine analgesia after gynecology tumor operation: a randomized trial of parecoxib used in postsurgical pain management. *The Journal of Surgical Research*.

[15] Fu W., Yao J., Li Q. (2014). Efficacy and safety of parecoxib/phloroglucinol combination therapy versus parecoxib monotherapy for acute renal colic: a randomized, double-blind clinical trial. *Cell Biochemistry and Biophysics*.

[16] Lamplot J. D., Wagner E. R., Manning D. W. (2014). Multimodal pain management in total knee arthroplasty: a prospective randomized controlled trial. *The Journal of Arthroplasty*.

[17] Kelley T. C., Adams M. J., Mulliken B. D., Dalury D. F. (2013). Efficacy of multimodal perioperative analgesia protocol with periarticular medication injection in total knee arthroplasty: a randomized, double-blinded study. *The Journal of Arthroplasty*.

[18] Murphy T. P., Byrne D. P., Curtin P., Baker J. F., Mulhall K. J. (2012). Can a periarticular levobupivacaine injection reduce postoperative opiate consumption during primary hip arthroplasty?. *Clinical Orthopaedics and Related Research*.

[19] Dirks J., Fredensborg B. B., Christensen D., Fomsgaard J. S., Flyger H., Dahl J. B. (2002). A randomized study of the effects of single-dose gabapentin versus placebo on postoperative pain and morphine consumption after mastectomy. *Anesthesiology*.

[20] Tröster A., Sittl R., Singler B., Schmelz M., Schüttler J., Koppert W. (2006). Modulation of remifentanil-induced analgesia and postinfusion hyperalgesia by parecoxib in humans. *Anesthesiology*.

[21] Chen D. W., Hsieh P.-H., Huang K.-C., Hu C.-C., Chang Y.-H., Lee M. S. (2010). Continuous intra-articular infusion of bupivacaine for post-operative pain relief after total hip arthroplasty: a randomized, placebo-controlled, double-blind study. *European Journal of Pain*.

